# Interface-engineered MoS_2_ heterostructures: from construction strategies to energy and photovoltaic applications

**DOI:** 10.1039/d5ra08711b

**Published:** 2026-03-09

**Authors:** Linhou Cong, Zixuan Yan, Siyu Chen, Peijin Yang, Weisheng Yang

**Affiliations:** a Kunming Institute of Physics Kunming 650221 China 15173277262@163.com ywsh@126.com; b Yunnan North Optical Technology Co., Ltd Kunming 650214 China

## Abstract

Two-dimensional MoS_2_ is a versatile semiconductor for optoelectronic and energy technologies, yet device performance is often constrained not by intrinsic layer properties but by interfacial bottlenecks such as energy-level misalignment, inefficient charge transfer, trap-mediated losses, and contact resistance. Recent progress in MoS_2_-based heterostructures demonstrates that nominal band diagrams alone are insufficient to predict outcomes; instead, device metrics emerge from a coupled interplay of energy-landscape reconstruction *via* interfacial dipoles and built-in fields, kinetic competition among charge transfer, recombination and trapping (*k*_CT_/*k*_rec_/*k*_trap_), and parasitic or contact limitations. Building on this mechanism-to-metrics view, this review summarises scalable construction strategies for vertical, lateral and mixed-dimensional MoS_2_ heterostructures, and organises interface types as actionable design levers spanning band-alignment classes, contact archetypes and bonding motifs. We further formalise a “backward design” route that starts from the target figure of merit, translates it into experimentally verifiable interfacial requirements including band offsets, dipole steps, PL/TA signatures, *R*_ct_ and contact resistivity, and then selects material pairing and geometry accordingly. To improve comparability beyond case-by-case reporting, a function–coupling–pairing summary and a minimum measurement checklist are provided. Photovoltaic and energy-storage case studies illustrate how Type-II alignment plus built-in fields suppress recombination and enhance extraction, while ion-permeable, strain-accommodating, Fermi-level-tuned interfaces accelerate charge-storage kinetics and stabilise cycling. Finally, we highlight remaining challenges in wafer-scale defect control, quantitative interface metrology, long-term stability and encapsulation, and interoperable data reporting toward manufacturable MoS_2_ heterostructure technologies.

## Abstract 2

Device metrics in MoS_2_ heterostructures are governed by interfacial energy-landscape reconstruction, rate competition (*k*_CT_/*k*_rec_/*k*_trap_) and contact limits. A backward-design map links measurable interface descriptors to practical engineering levers.

## Graphical abstract

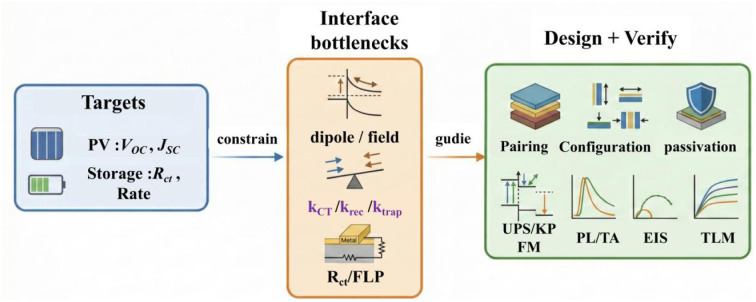

## 1 Introduction

Two-dimensional transition-metal dichalcogenides have reshaped low-dimensional electronics and energy devices, and MoS_2_ has become a widely adopted semiconductor platform owing to its gate-tunable transport, strong light–matter interaction and chemically addressable surface.^1,2^ These attributes underpin a broad range of concepts from photodetectors and photovoltaic junctions to electrocatalysis and electrochemical energy storage.^3,4^ However, a recurring lesson is that practical figures of merit—efficiency and photovoltage, response speed, hysteresis, stability and device-to-device variability—are frequently governed by interfaces and contacts rather than by the intrinsic MoS_2_ channel itself.^5^

Over the past decade, MoS_2_-based heterostructures have rapidly expanded the design space by integrating MoS_2_ with diverse partners, including other two-dimensional crystals, three-dimensional semiconductors, oxides and organics.^6–8^ Vertical van der Waals stacks, lateral in-plane junctions and mixed-dimensional hybrids have enabled deliberate tuning of band offsets, interfacial fields and carrier pathways, while advances in scalable growth and transfer have improved material quality and device integration. As a result, MoS_2_ heterostructures have demonstrated increasingly competitive performance in optoelectronics and energy-related applications, and have highlighted interface engineering as a primary route to functional enhancement.^8–10^

Despite this progress, predictability and cross-study comparability remain limited. Nominal band alignment (Type-I/II/III) often fails to capture real interfacial energy reconstruction induced by dipoles, built-in fields and dielectric screening, and process-induced non-idealities such as residues, adsorbates, vacancies, grain boundaries and strain can introduce traps, potential fluctuations and parasitic leakage channels.^6,8,14^ In parallel, metal/MoS_2_ contacts commonly suffer from Fermi-level pinning and large contact resistance, which can impose an upper bound on extraction and speed even when the junction design is favourable.^15–17^Consequently, devices based on nominally similar heterojunctions may be limited by different rate-determining segments, and performance trends are difficult to generalise.

The purpose of this review is to translate the rich but fragmented MoS_2_ heterostructure literature into an actionable decision framework that links material pairing to interfacial coupling and ultimately to device function. We adopt a mechanism-to-metrics view in which device outcomes are determined by the coupled competition among interfacial charge transfer, recombination and trapping (*k*_CT_/*k*_rec_/*k*_trap_), together with contact and parasitic limitations. Based on this perspective, we formalise a “backward design” route that starts from target figures of merit, translates them into experimentally verifiable interfacial requirements (band offsets and barriers, dipole steps and built-in fields, transient signatures, interfacial charge-transfer resistance *R*_ct_ and contact resistivity), and then guides the selection of pairing and configuration (vertical, lateral or mixed-dimensional) and the corresponding interface-engineering levers. To improve comparability beyond case-by-case reporting, we provide a function-coupling–pairing summary and a minimum measurement checklist, and illustrate the framework through photovoltaic and energy-storage case studies.

## 2 Physicochemical properties of MoS_2_ and strategies for constructing MoS_2_-based heterostructures

### 2.1 Intrinsic physicochemical properties: lattice and band structure

Before discussing the construction methods and interfacial regulation mechanisms of MoS_2_ heterostructures, it is essential to understand their intrinsic crystal structures and phases. The lattice characteristics of MoS_2_ directly determine its growth mode in heterojunctions, the distribution of interfacial stresses and the behaviour of energy-band alignment.

**Fig. 1:**
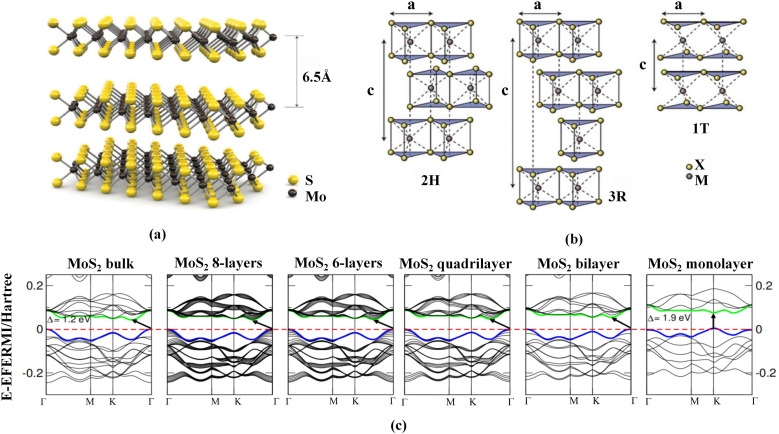
Lattice structure and band diagram of MoS_2_. (a) Layered MoS_2_ structure along the *c*-axis, with an interlayer spacing of approximately 6.5 Å.^11^ Adapted from ref. 11 with permission from Springer Nature, copyright 2011. (b) Common polytypes of MoS_2_, including the 2H, 3R, and 1T phases.^12^ Adapted from ref. 12 with permission from Springer Nature, copyright 2012. (c) Evolution of the band structure from bulk to monolayer, with the bandgap transitioning from an indirect bandgap (≈1.2 eV) to a direct bandgap (≈1.9 eV).^13^ Adapted from ref. 13 with permission from the American Physical Society, copyright 2011.

The lattice structure and band diagram of MoS_2_ are shown in Fig. 1. As a typical layered transition-metal dichalcogenide, MoS_2_ belongs to the hexagonal crystal system and is composed of S–Mo–S triatomic layers, where the Mo atom is surrounded by six S atoms to form an X–M–X sandwich structure (Fig. 1(a)). In the crystal, these monolayers are stacked along the *c*-axis by van der Waals forces to form multilayer materials with an interlayer spacing of approximately 6.5 Å.^11^ MoS_2_ exists in several crystal phases, among which the thermodynamically stable 2H phase is most common, featuring trigonal-prismatic coordination of Mo. In addition, there are 3R-stacked isomorphic variants and a metallic 1T phase (Fig. 1(b)).^12^ These different stacking configurations and coordination environments lead to marked differences in symmetry and electronic structure, providing an important physical basis for constructing both homogeneous and heterogeneous heterostructures.

In addition to structural diversity, the electronic structure of MoS_2_ also shows a pronounced response to the number of layers (Fig. 1(c)). From bulk to monolayer, the bandgap of MoS_2_ gradually evolves from an indirect bandgap to a direct bandgap, with the value increasing from approximately 1.2 to 1.9 eV. In bulk and multilayer MoS_2_, the valence-band maximum and conduction-band minimum are located at the *Γ* and *K* points, respectively; in the monolayer, both are located at the *K* point, forming a direct bandgap that is significant for enhancing light absorption and exciton binding.^13^ The tunability of both the structure and the electronic states endows MoS_2_ with excellent structural compatibility and band-engineering flexibility for heterostructure construction.

### 2.2 Structural classifications: vertical, lateral, and hybrid configurations

Building on the physicochemical characteristics and band-structure fundamentals of MoS_2_, MoS_2_-based heterostructures can be conveniently organised according to interfacial dimensionality and bonding nature into three recurring configurations: vertical (van der Waals stacked), in-plane (or lateral, laterally stitched), and hybrid (often mixed-dimensional). This taxonomy is not purely morphological; it pre-defines the dominant cross-interface transport mode (out-of-plane tunnelling/drift *versus* in-plane diffusion, or their combination), and it constrains the attainable interfacial potential gradients, strain accommodation, and defect tolerance. It therefore provides a unified entry point for the mechanistic discussions in later sections and for device-oriented material pairing and integration strategies.

**Fig. 2:**
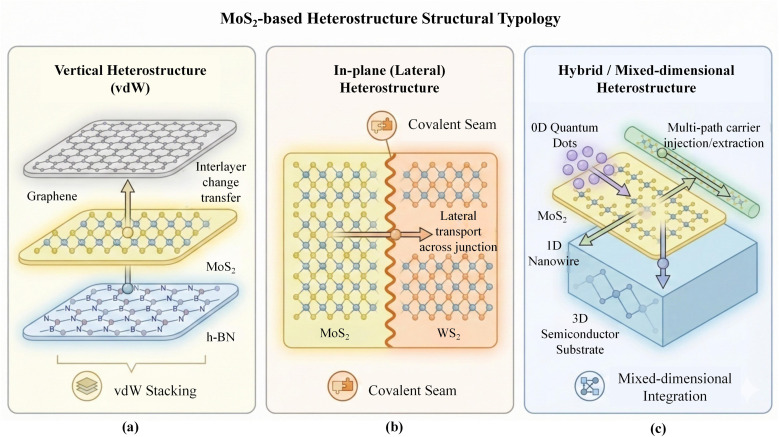
Structural typology of MoS_2_-based heterostructures. (a) Vertical van der Waals (vdW) heterostructure constructed by face-to-face stacking (*e.g.*, graphene/MoS_2_/h-BN), enabling interlayer charge/exciton transfer. (b) In-plane (lateral) heterostructure featuring an atomically stitched covalent seam, supporting lateral carrier transport across the junction (*e.g.*, MoS_2_–WS_2_). (c) Hybrid/mixed-dimensional heterostructure integrating 2D-MoS_2_ with 0D/1D/3D components to provide multi-path carrier injection/extraction. Arrows indicate representative schematic transport pathways rather than quantitative fluxes.

For 2D/2D systems, vertical and in-plane architectures represent two complementary extremes in junction geometry and pathway topology. In vertical heterostructures, MoS_2_ is stacked face-to-face with graphene, h-BN, or other transition-metal dichalcogenides (TMDs) to form an area-contact junction separated by an Å-scale van der Waals gap, making band offsets and interlayer charge-transfer characteristics broadly designable (Fig. 2(a)).^18–20^ The weak interlayer bonding largely relaxes lattice-matching requirements and enables atomically flat interfaces across diverse material combinations.^20,21^ As a trade-off, performance can become highly sensitive to interfacial cleanliness and relative crystallographic orientation (*e.g.*, twist angle), which modulate tunnelling probability and the accessible transfer channels; accordingly, fabrication routes span transfer-and-stack assembly as well as direct-growth/epitaxial approaches. Representative studies have shown that vertical stacks such as MoS_2_/WS_2_ can form effective Type-II heterojunctions and exhibit ultrafast interlayer charge separation and exciton dynamics, highlighting the advantage of vertical architectures for photocarrier extraction and recombination regulation.^22,23^ In contrast, in-plane (lateral) heterostructures emphasise edge-to-edge epitaxy and covalent stitching within the same two-dimensional lattice, forming an atomically sharp one-dimensional interface (a seam) (Fig. 2(b)).^24,25^ This geometry introduces lateral band offsets and built-in electric fields within monolayer/few-layer sheets, and the interfacial potential landscape can be accessed more directly by planar electrodes without additional interfacial resistance associated with interlayer tunnelling. However, the growth window is typically narrower, imposing stricter requirements on precursor switching, edge reactivity, and defect control at the junction, because boundary states and seam “sharpness” directly translate into leakage and recombination losses. WS_2_–MoS_2_ in-plane heterostructures realised *via* one-step or sequential growth have exhibited experimentally observable lateral built-in potentials, underscoring their distinct advantages for lateral junction devices and in-plane integration. Subsequent studies on laterally stitched TMDCs further emphasised the role of atomically stitched seams in band-structure tuning as well as contact/interface engineering.

Hybrid heterostructures in the literature usually carry two closely related meanings (Fig. 2(c)). One refers to systems that combine vertical and in-plane elements within a single platform (*e.g.*, multi-material vertical stacks incorporating localised lateral junctions). More commonly, “hybrid” denotes mixed-dimensional (2D + *n*D) architectures, in which 2D MoS_2_ is integrated with 0D quantum dots, 1D nanotubes/nanowires, or 3D semiconductors such as Si and GaN.^26–28^ Such integration expands the optical absorption cross-section, facilitates carrier separation/injection, and can also provide additional degrees of freedom for interfacial strain relaxation. Jariwala *et al.* systematically reviewed mixed-dimensional van der Waals heterostructures and pointed out that the primary challenges lie in interfacial state passivation, band-alignment stability, and process uniformity over large areas.^29^ Recent perspective articles on lateral heterostructures have further highlighted that structural dimensionality and interfacial “sharpness” are likely to be key variables governing the tunability of excitons and other quasiparticles, reinforcing the practical importance of configuration-level classification before moving to detailed mechanism discussions.

### 2.3 Scalable construction strategies

To achieve high-performance MoS_2_-based heterostructures, various construction methods have been employed, including chemical vapour deposition (CVD), transfer assembly and solution-based routes. Each method plays a crucial role in synthesising different types of heterostructures. Owing to its ability to provide precise and controllable material growth together with suitability for large-scale production, CVD is the most widely used approach for MoS_2_-based heterostructures.

Gong first introduced a one-step CVD method for fabricating MoS_2_/WS_2_ lateral heterostructures by controlling the temperature-zone arrangement of WO_3_ and MoO_3_ precursors.^23^ This enabled sequential growth of MoS_2_ and WS_2_, forming an atomically connected heterojunction. Transmission electron microscopy and energy-dispersive spectroscopy confirmed that the interfacial transition region was 4–6 nm with minimal lattice mismatch, demonstrating excellent epitaxial stitching capability. This strategy has since been extended to other MoS_2_-based systems, such as MoS_2_/MoSe_2_ and MoS_2_/WSe_2_, achieving continuous epitaxial growth by controlling the S/Se ratio, heating rate and temperature zones.^33–35^ Additionally, for heterojunctions involving dissimilar materials, the one-step route together with catalytic substrates or oxygen control facilitates interfacial connection and charge transfer, making it suitable for optoelectronic detection and bandgap tuning.^36,37^ It also enables regionally controlled phase-transition growth in homogeneous materials, such as 1T-MoS_2_ and 2H-MoS_2_, thereby enhancing charge separation and catalytic activity.^38,39^

Compared with the one-step CVD method, the two-step CVD route allows initial growth of one material followed by epitaxial growth of another at the edges or on the surface. This enables heterostructures with larger lateral size, more complex architectures, clearer interfaces and richer material combinations. In 2020, a two-step CVD strategy was reported to fabricate a high-quality metal–semiconductor NiTe_2_/MoS_2_ heterostructure that exhibited excellent electronic and optoelectronic properties, attributed to a more ideal heterojunction interface.^40^ In 2022, related work achieved controlled growth of both lateral and vertical MoS_2_/ReS_2_ heterojunctions using the two-step method, revealing the relationship between interfacial structure and growth parameters.^41^ Furthermore, a 2023 study employed the two-step method to synthesise MoS_2_/MoSe_2_ heterostructures with *in situ* growth that avoided mechanical transfer, yielding cleaner interfaces and tunable physical properties.^42^ These advances highlight the greater freedom and controllability of the two-step route in material selection and structural design, making it suitable for constructing complex stacks such as multilayer encapsulations or superlattice-like configurations.

**Fig. 3:**
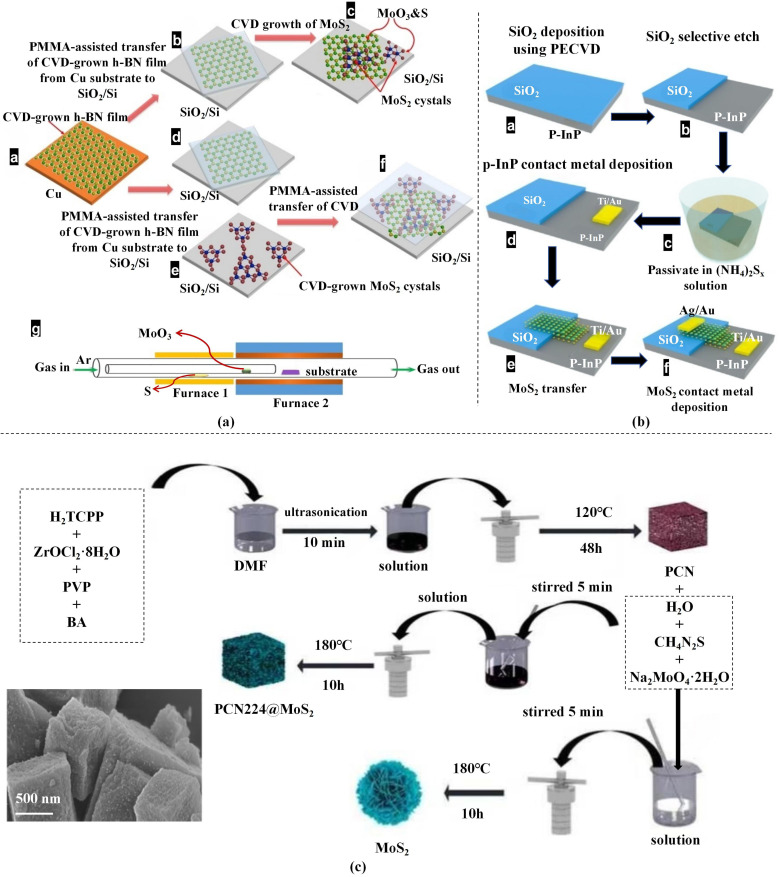
Schematic illustrations of MoS_2_-based heterostructures fabricated by transfer strategies and the CVD method. (a) CVD and dry-transfer routes for fabricating MoS_2_/h-BN heterostructures, where panels (a–c) show MoS_2_/h-BN prepared by CVD, panels (a, d–f) employ mechanical exfoliation to assemble MoS_2_/h-BN, and (g) presents the process flow for CVD-based MoS_2_/h-BN fabrication.^30^ Adapted from ref. 30 with permission from the American Chemical Society, copyright 2015. (b) Process flow diagram for MoS_2_/p-InP heterostructures *via* wet transfer.^31^ Reproduced from ref. 31 under a Creative Commons Attribution (CC BY) license. (c) Schematic illustration of the solvothermal synthesis of PCN-224/MoS_2_ heterostructures and a representative SEM image of the PCN-224/MoS_2_ heterostructures.^32^ Adapted from ref. 32 with permission from Elsevier, copyright 2024.

In addition to conventional CVD, alternative approaches such as transfer strategies and solvothermal routes have been explored for fabricating MoS_2_-based heterostructures. Fig. 3 presents schematic illustrations of MoS_2_-based stacks realised by transfer strategies and CVD. Transfer techniques, including dry (Fig. 3(a)) and wet methods (Fig. 3(b)), are widely used; dry transfer typically offers better interfacial quality and is suitable for small-area, high-quality production.^43–45^ The solvothermal method, which performs high-temperature and high-pressure reactions in organic solvents, is simple, highly controllable and amenable to large-scale production (Fig. 3(c)). It has been widely applied to heterostructures with semiconductors such as CdIn_2_S_4_, CdS and TiO_2_, especially for photocatalytic hydrogen evolution and pollutant degradation.^46–48^ Other techniques, including ALD, MBE and mechanical exfoliation, provide additional options for MoS_2_-based heterostructures with tunable properties for diverse applications.^49,50^

In tandem with the development of diverse fabrication strategies, considerable efforts have focused on optimising the interfacial quality and scalability of MoS_2_-based heterostructures. Because the physical and chemical properties of heterostructures are strongly influenced by interface cleanliness, lattice mismatch and defect density, the choice of fabrication technique plays a decisive role in determining device performance in optoelectronic, catalytic and energy-related applications. For example, transfer-based methods are frequently combined with surface treatments or encapsulation layers to mitigate interfacial contamination, whereas solvothermal synthesis leverages precise precursor regulation to fine-tune the morphology and composition of hybrid systems. Furthermore, combining bottom-up techniques with top-down approaches has proven effective for integrating high-quality monolayers into complex device architectures, as exemplified by GQD/MoS_2_ and BiVO_4_/MoS_2_ heterostructures.^31,32^ Such hybrid strategies not only enhance reproducibility and structural uniformity but also provide versatile routes for engineering heterostructures with multifunctional properties, thereby facilitating their incorporation into emerging nanoelectronic and photonic systems.

## 3 The type of constituent materials and interface engineering in MoS_2_-based heterostructures

**Fig. 4:**
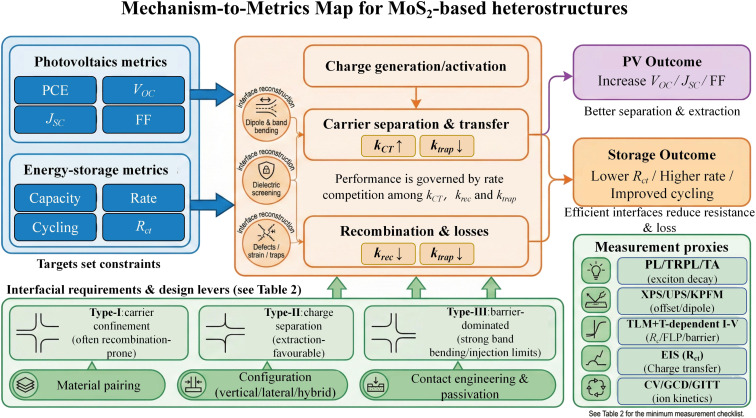
Mechanism-to-metrics map for MoS_2_-based heterostructures. Target device metrics for photovoltaics (PCE, *V*_OC_, *J*_SC_, FF) and energy storage (capacity, rate, cycling, *R*_ct_) constrain interfacial requirements that can be expressed as a competition among charge-transfer, recombination and trap-assisted loss rates (*k*_CT_, *k*_rec_, *k*_trap_). Interfacial reconstruction (dipoles/band bending, dielectric screening, and defects/strain/traps) reshapes barrier heights and built-in fields, thereby governing carrier separation/transfer and loss pathways and determining the resulting PV and storage outcomes. Practical measurement proxies and a minimum checklist (Table 2) are highlighted to keep the framework experimentally actionable for material pairing, configuration selection and contact engineering.

Following Chapter 2, this chapter develops an interface-centred framework linking MoS_2_-based heterojunction design to measurable device performance. Fig. 4 outlines the core logic: target metrics constrain interfacial requirements, which can be expressed as a competition among charge-transfer, recombination and trap-assisted loss rates governed by the effective interfacial energy landscape. Material choice sets feasible band offsets and alignment classes, whereas interfacial chemistry and geometry—dipoles and band bending, defects/traps, dielectric screening, contact effects and strain compatibility—determine barrier heights, built-in fields and the resulting transport and loss pathways. The discussion is anchored to practical measurement proxies Fig. 4 and a minimum checklist (Table 2), providing design guidance for the application-oriented chapters that follow.

### 3.1 Common types of constituent materials in MoS_2_-based heterostructures

The selection of appropriate constituent materials is crucial for the performance optimisation of MoS_2_-based heterostructures. Table 1 presents the main characteristics of the different material systems, including their band alignment with MoS_2_ and the direction of dominant carrier transport. The combination of materials not only determines the electronic structure of MoS_2_ but also profoundly affects performance characteristics such as interfacial charge transfer, optoelectronic response and catalytic efficiency. The interactions between different materials, particularly the band alignment and the carrier-transfer direction, dictate the performance of the heterojunctions.

**Table 1:** MoS_2_-based heterojunction reference table

Material systems	Representative materials	Band alignment/contact	Transport mechanism	References
2D semiconductors	WS_2_, WSe_2_, ReS_2_, MoSe_2_, HfS_2_, InSe, GaTe, SnSe_2_, black phosphorus (BP)	Majority: Type II	e^−^: to MoS_2_	51–59
		Minority: Type I (controlled transformation)	h^+^: to opposite layer	
2D metals and semi-metals	Graphene, PtSe_2_ (semimetal), MXene (Ti_3_C_2_T_*x*_)	Majority: Schottky (n-type), Fermi-level pinning	e^−^: injection to MoS_2_	60–62
		Minority: near-ohmic *via* vdW contact + work-function/doping/interlayers	h^+^: injection difficult; improved with high-WF + p-doping	
2D insulators	h-BN	Type I (tunnelling/capacitive spacer)	Carriers mainly in MoS_2_; under bias, e^−^/h^+^ tunnel across h-BN to the opposite side	63 and 64
2D magnetic and ferroelectric functional layers	CrBr_3_, NiTe_2_, In_2_Se_3_, GeSe	Magnetic: Type II (majority)	Mag./FE insulators: carriers localised in MoS_2_	40 and 65–67
		Ferroelectric: Type I/II/III (minority, polarisation-dependent)	Mag. semiconductor: e^−^ → MoS_2_, h^+^ → opposite layer	
			Mag. metal: e^−^ → MoS_2_FE semiconductor: e^−^/h^+^ → MoS_2_ (polarisation-controlled)	
3D conventional semiconductors	4H-SiC, GaN, AlN	Majority: Type II	e^−^: to MoS_2_	68–70
		Minority: Type I/Type III	h^+^: to 3D semiconductor	
Oxides/transparent conductors/dielectrics	TiO_2_, WO_3_, ZnO, SrTiO_3_, TCOs (AZO), Al_2_O_3_	n-type oxides: Type II (majority)	n-type oxides: e^−^ → MoS_2_, h^+^ → oxide	71–76
		Dielectrics: insulating spacer	Dielectrics: carriers mainly in MoS_2_ (field modulation)	
		TCOs: Schottky-like contact	TCOs: carrier extraction *via* TCO contact	
Halide perovskites	MAPbI_3_, CsPbBr_3_	Majority: Type II (perovskite/MoS_2_)	e^−^: To MoS_2_	77 and 78
		Minority: possible reversal or alignment transition	h^+^: to perovskite/opposite layer	
Organic semiconductors/small-molecule polymers	C_60_, rubrene (organic semiconductor)	Majority: Type II for p-type donor/fullerene acceptor with n-type MoS_2_	e^−^: to MoS_2_	79–82
			h^+^: to organic layer	
Zero-/one-dimensional nanomaterials	Quantum dots (PbS, CdSe), carbon nanotubes (CNTs), silver nanowires (Ag NWs)	Majority: Type II	e^−^: to MoS_2_	83–85
		Metal nanowires/MoS_2_: Schottky or near-ohmic contact	h^+^: to opposite component	
Metal electrodes/work-function engineering	Au, Pt, Pd, Ti, Ni, Ag	Most metals/MoS_2_: tunable n-type Schottky contacts	Most metals/MoS_2_: e^−^ → MoS_2_ (predominant)	86–90
		High-work-function + p-type doping/dipoles/phase: near-ohmic possible	High-work-function + p-type doping/dipoles/phase: h^+^ → MoS_2_ (predominant)	

When two-dimensional semiconductor materials are combined with MoS_2_, they typically exhibit Type-II band alignment, which facilitates the effective separation and transfer of electrons and holes and thereby enhances optoelectronic effects. Metal and semimetal materials form Schottky contacts with MoS_2_, influence the direction of electron injection and adjust the contact characteristics of devices. In contrast, insulators and dielectric materials provide electrical isolation at the interface, which helps improve the stability of MoS_2_-based heterostructures, reduces interface defects and optimises device performance. Functional materials such as magnetic, ferroelectric and perovskite systems not only contribute to conventional optoelectronic effects but also optimise MoS_2_-based devices by modulating spin and polarisation effects. Therefore, the fine-tuned selection and combination of materials are key to enhancing the performance of MoS_2_-based heterostructures in the future.

### 3.2 Interfacial coupling mechanisms and device-limiting factors in MoS_2_-based heterojunctions

**Fig. 5:**
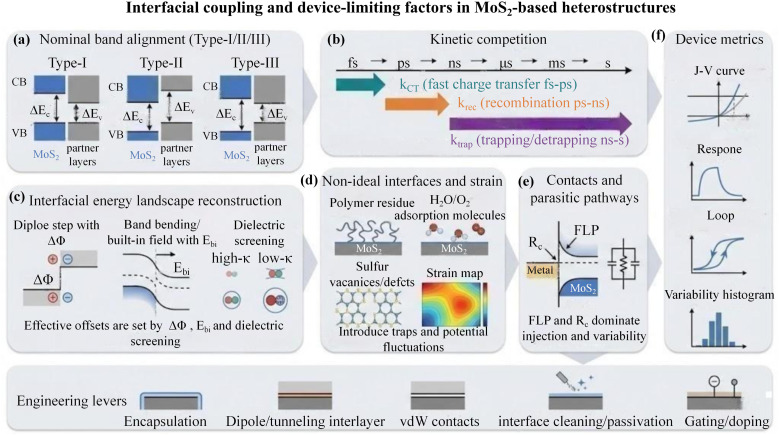
Interfacial coupling and device-limiting factors in MoS_2_-based heterostructures. (a) Nominal Type-I/II/III band alignment. (b) Kinetic competition among charge transfer (*k*_CT_), recombination (*k*_rec_) and trapping (*k*_trap_). (c) Interfacial energy-landscape reconstruction (dipole step Δ*Φ*, built-in field *E*_bi_, dielectric screening). (d) Non-ideal interfaces/strain (residues, adsorbates, vacancies) creating traps and potential fluctuations. (e) Contact/parasitic limitations (FLP, *R*_c_, leakage/shunts). (f) Device outputs: *J*–*V*, transient response, hysteresis, and variability; bottom icons indicate main engineering levers (encapsulation, interlayers, vdW contacts, cleaning/passivation, gating/doping).

Section 3.1 summarised how material pairing defines the nominal band-alignment types in MoS_2_-based heterojunctions. In practical devices, however, performance is rarely a linear superposition of the intrinsic properties of each layer. Instead, it is more often governed by interfacial coupling at and around MoS_2_, including charge redistribution, interfacial energy-landscape reconstruction, and transfer–recombination kinetics. For instance, Chen and co-workers observed efficient interfacial charge transfer and the formation of interlayer excitonic states in atomically thin MoS_2_/WS_2_ van der Waals heterostructures, indicating that device outputs are frequently limited by interfacial transfer and recombination dynamics rather than by a simple additive combination of single-layer properties.^91^ Accordingly, this section is not organised by partner material classes or stacking geometries. Instead, it consolidates recurring interfacial-physics modules across MoS_2_-based heterostructures and clarifies why an apparently favourable nominal alignment can still translate into device bottlenecks. Configuration-specific effects associated with vertical, lateral, and mixed-dimensional architectures are deferred to Section 3.3. Fig. 5 provides a schematic map linking these interfacial modules to device-level metrics.

In MoS_2_-based heterojunctions, the effective interfacial energy landscape is often “rewritten” upon contact, rather than being a static band diagram assembled from isolated constituents. Nominal band offsets Δ*E*_c_/Δ*E*_v_ describe the thermodynamic driving force for charge separation, yet interfacial dipoles can introduce a vacuum-level step and shift band edges away from values inferred from isolated-layer energetics. This behaviour has been quantitatively verified in mixed-dimensional heterojunctions such as MoS_2_/GaN(0001), where Henck and co-workers combined ARPES with high-resolution XPS to identify an interfacial dipole of approximately 0.2 eV and to show that contact-induced charge redistribution can substantially modify the effective band structure.^92^ After contact formation, Fermi-level equilibration drives charge redistribution and local band bending, yielding a built-in field that can be further tuned by electrostatic gating and interfacial charge-transfer doping. Dielectric screening from encapsulation or nearby high-*k* environments can renormalise Coulomb interactions, alter the apparent bandgap and exciton binding energy, and make the “effective” alignment strongly environment-dependent. Consistent with this picture, Ryou and co-workers systematically compared different dielectric environments and reported that the bandgap of monolayer MoS_2_ can vary markedly (approximately from 2.8 eV to 1.9 eV), underscoring that effective energy levels and barrier profiles in two-dimensional systems are not fixed constants but can be reshaped by the surrounding screening conditions.^93^ These coupled effects explain why a nominal Type-II alignment does not necessarily yield the expected barrier profile or built-in field under operating conditions.

Whether a reconstructed landscape produces measurable output is controlled by kinetic competition at the interface. For optoelectronic operation, efficient separation requires the interfacial charge-transfer rate *k*_CT_ to outpace intrinsic recombination *k*_rec_ and trapping *k*_trap_. The accessible transfer channels and *k*_CT_ depend sensitively on interlayer distance, coupling strength, twist angle, and momentum-space matching. Ultrafast spectroscopy provides direct quantitative evidence for this sensitivity, as Zimmermann and co-workers investigated twist-angle-dependent MoS_2_/WSe_2_ heterostructures using time- and polarisation-resolved second-harmonic imaging pump–probe microscopy, resolving electron transfer from WSe_2_ to MoS_2_ on sub-20 fs timescales with the fastest component reaching ∼12 fs.^94^ Valley mismatch commonly introduces phonon-assisted pathways, slowing transfer and increasing exposure to recombination or trapping. Following separation, long-lived interlayer excitons or spatially separated states can raise collection probability, but when defect-assisted pathways dominate, such extended lifetimes can also manifest as photogating, current tails, elevated noise, and response hysteresis. Efficiency, speed, and noise therefore emerge from a shared interfacial rate hierarchy rather than from independent optimisation knobs.

Non-ideal interfaces further convert the problem from a single-barrier picture into a trap-dominated, multi-channel landscape. Transfer residues, adsorbed H_2_O/O_2_, sulfur vacancies, and grain boundaries introduce charged scatterers and deep states that amplify potential fluctuations, promote (local) Fermi-level pinning, and degrade mobility, stability, and device-to-device uniformity.^15,16^ Di Bartolomeo and co-workers systematically analysed transfer-curve hysteresis in MoS_2_ devices and showed that the hysteretic behaviour is closely linked to interfacial charge trapping processes, with contributions from surface adsorbates (H_2_O/O_2_) and intrinsic defects such as sulfur vacancies.^95^ Because two-dimensional channels lack bulk dielectric screening, even subtle environmental perturbations can yield pronounced hysteresis, threshold drift, and slow transients. Under illumination, capture–release kinetics frequently sets the observed time constants. Strain and stress are often entangled with defect formation and interfacial charging, reshaping barrier profiles and local fields, so treating “defects–strain–electrostatics” as a coupled set is typically more predictive than considering each factor in isolation.

In many MoS_2_-based devices, the dominant bottleneck arises not at the heterojunction itself but at contacts and parasitic pathways. Metal/MoS_2_ interfaces commonly exhibit Schottky barriers and high contact resistance *R*_c_, driven by metal-induced gap states together with defect states that enforce strong Fermi-level pinning, which limits the effectiveness of work-function engineering alone. In this context, Kim and co-workers emphasised that suppressing metal-induced gap states is a key route to reducing pinning and restoring barrier tunability, providing a mechanistic rationale for why contact engineering is often more effective than simply changing the contact metal.^96^ Parasitic conduction through non-junction regions, edge leakage, and local shunt paths can dilute rectification or photovoltaic contributions and inflate variability. Wi and co-workers likewise highlighted that pinholes or edge-related leakage defects can form unintended leakage channels and reduce the open-circuit voltage, implying that parasitic pathways may mask the true contributions of interfacial barriers and built-in fields.^97^ As a result, experimentally measured device metrics—efficiency/photovoltage, transient response speed, stability/hysteresis, and variability—often reflect a convolution of interfacial reconstruction, kinetic competition, and injection/leakage conditions.

MoS_2_-based heterojunctions are therefore best rationalised within a unified framework of energy-landscape reconstruction, kinetic competition, and non-ideal/contacts limitations. From a device-engineering perspective, performance targets are more reliably approached by back-tracing to interfacial descriptors—band offsets, dipole steps, built-in fields, the *k*_CT_/*k*_rec_ hierarchy, acceptable trap densities, and upper bounds on *R*_c_—and then implementing dielectric/encapsulation control, interfacial cleaning and passivation, dipole or tunnelling interlayers, van der Waals contacts, and gating/doping. This mechanism-to-metrics linkage also sets the physical basis for Section 3.3, where configuration-dependent degrees of freedom are discussed.

### 3.3 How configuration maps generic interfacial mechanisms onto device pathways and performance trade-offs

**Fig. 6:**
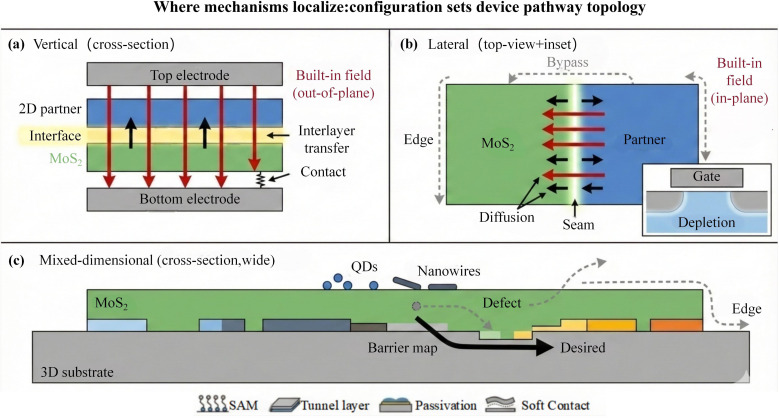
Where mechanisms localize: configuration sets device pathway topology in MoS_2_-based heterostructures. (a) Vertical (vdW stacked) devices localize key processes to an interfacial thin region and often become contact-limited. (b) Lateral devices localize key processes to a one-dimensional seam and are sensitive to bypass/edge leakage, where geometry and gating can suppress non-junction conduction. (c) Mixed-dimensional devices are governed by spatially inhomogeneous interfacial barriers (“barrier map”), where defect-assisted and edge-related bypass paths can amplify device-to-device variability; interfacial engineering tools (*e.g.*, SAMs, ultrathin tunnel layers, passivation, and soft/vdW contacts) are commonly used to stabilize the effective pathway.

This section adopts a structure-determines-pathway viewpoint to explain how different configurations “place” the generic interfacial mechanisms summarised in Section 3.2 into specific spatial regions and transport channels. Rather than reiterating the definitions of interfacial dipoles, energy-landscape reconstruction, or kinetic competition, we focus on device-facing questions: where the generic mechanisms localise, how carriers are routed along the effective pathway, and where bypass channels emerge and set upper bounds on device outputs. Across three representative configurations—vertical stacking, lateral in-plane junctions, and mixed-dimensional architectures—this configuration-to-pathway-to-output mapping has been repeatedly emphasised in MoS_2_-based heterostructure studies and reviews, and it is increasingly shaping application-oriented configuration selection. Fig. 6 provides a side-by-side schematic comparison of the localisation of mechanisms, dominant transport pathways, and typical bypass routes under the three configurations, enabling a unified discussion of pathway topology and the corresponding limiting factors.

In the vertical configuration, the heterointerface is an area contact with an Å-scale interlayer gap, such that the potential drop and built-in field are most naturally established along the out-of-plane direction, and cross-junction transport is dominated by short-range interlayer tunnelling or drift, as illustrated in Fig. 6(a). Under this geometry, decisive processes are highly confined within an ultrathin interfacial zone: interfacial cleanliness, insertion-layer thickness windows, and the spatial distribution of interfacial traps collectively determine whether the effective pathway can be constrained to the intended interlayer channel. Samad *et al.* realised controllable CVD growth of vertical heterostructures comprising monolayer and multilayer MoS_2_, providing a clear structural example consistent with interfacial-zone-dominated transport in vertical architectures.^98^ Importantly, many vertical devices are not limited solely by the junction itself; the electrode readout side can become the system bottleneck, with contact resistance imposing an upper bound on usable bandwidth. Andrews *et al.*.outlined contact-engineering routes to reduce the contact barrier and contact resistance in MoS_2_ transistors, underscoring that vertical design priorities should front-load both interfacial-channel confinement and contact/readout optimisation in order to translate intrinsically fast interlayer processes into device-level performance.^99^

In the lateral in-plane configuration, the heterojunction is compressed into a one-dimensional seam, the built-in field is primarily in-plane, and separation/collection depend more strongly on diffusion length, junction geometry, and boundary states, as schematically shown in Fig. 6(b). Here, the localisation of generic mechanisms shifts from an interfacial thin zone to the seam and its vicinity; boundary defects, band-edge roughness, and locally non-uniform barriers can directly affect the effective pathway length and the fraction of carriers that can be collected. Chen *et al.* quantitatively characterised the lateral built-in potential in monolayer MoS_2_–WS_2_ in-plane heterostructures, providing direct evidence that the in-plane field distribution is highly sensitive to the seam environment.^100^ At the device level, lateral architectures are also more prone to geometry-enabled bypass channels: non-junction parallel conduction and edge bypass routes can dilute the junction contribution, such that an apparently favourable nominal alignment does not necessarily yield strong rectification or photovoltaic output; Fig. 5(b) also indicates representative edge/non-junction bypass pathways and their suppression *via* electrostatic depletion. Wu *et al.* demonstrated self-driven photovoltaic photodetection based on monolayer MoS_2_–WS_2_ in-plane heterostructures, exemplifying the structural trade-off between seam-junction contribution and bypass suppression, and suggesting that geometric definition, gate-driven depletion, and boundary passivation often set the output ceiling prior to further material substitution.^101^

In mixed-dimensional architectures, MoS_2_ forms multi-scale contacts with bulk semiconductors or 0D/1D absorbers, and the effective pathway is governed less by a single interface or a single seam than by a spatially varying barrier distribution, as depicted in Fig. 6(c). Local oxides, interfacial reactions, patchy barrier heights, and defect-assisted channels can coexist, producing parallel transport pathways that amplify area effects and device-to-device variability. Dhyani *et al.* reported scalable Si/MoS_2_ p-n heterojunction photodetectors, which typify the interplay between cross-interface injection and parasitic channels in mixed-dimensional systems.^102^ In addition, fabrication studies on vertical MoS_2_/Si multilayer heterojunctions highlight practical challenges associated with interfacial-layer formation and barrier uniformity, reinforcing that mixed-dimensional designs must treat interfacial chemistry stability and barrier-statistics convergence as first-order engineering variables rather than inferring device outputs from nominal band offsets alone.^103^

Overall, the three configurations correspond to distinct choices of pathway topology and non-ideality localisation. Vertical architectures are well suited for high-bandwidth and fast response, but typically require stringent suppression of interfacial traps together with reduction of contact/readout ceilings. Lateral in-plane architectures are naturally compatible with planar integration, yet rely more heavily on geometry- and gating-driven suppression of parallel and edge bypass conduction. Mixed-dimensional architectures favour large-area manufacturability and process compatibility, but must treat interfacial chemistry and barrier uniformity/statistics as primary levers to control variability. In line with the backward-design theme of this review, a robust strategy is to first select the configuration to fix pathway topology, then translate the interfacial descriptors in Section 3.2 into target-driven constraints, and finally use geometry definition, insertion layers, passivation, and contact engineering to structurally exclude bypass channels, thereby enabling nominally favourable alignments to more reliably convert into reproducible device outputs.

### 3.4 Framework for rational interface engineering in MoS_2_-based heterostructures

This subsection, building on the interfacial mechanisms discussed in Sections 3.1–3.3, formalises a practical design route for MoS_2_-based heterostructures. The idea is to begin from the device-level objective, to express that objective as an interfacial requirement that can be measured, and only then to determine the material pairing and geometry that can realise it under realistic processing conditions. To make this route directly usable, Table 2 brings together several representative device classes (optoelectronic, photodetection, transistor/contact, electrocatalytic, and energy-storage or PEC systems). For each class it specifies the interfacial coupling that must be present, the MoS_2_-based heterostructure configurations that have been reported to provide it, and the set of key parameters that should be reported in order to prove that the intended interface has in fact been established. The application-oriented sections that follow can thus refer to Table 2 to situate published devices within the present framework and to compare different material systems on a consistent footing.

**Table 2:** Function-coupling–pairing summary for MoS_2_-based heterostructures

Function/KPI	Required interfacial coupling	MoS_2_-based pairing/geometry	Key parameters to be measured (minimum checklist)	Ref.
Photovoltaics/photoelectronic MoS_2_ junctions	Type-II (staggered) band alignment; built-in field; low-recombination interface (often trap/*R*_c_ limited)	MoS_2_/CdS thin-film junction; MoS_2_/Si or GaAs (2D–3D); vertical GaSe/MoS_2_; graded MoS_2*x*_Te_2(1−*x*)_/MoS_2_ vdW stack	Band offsets/junction type (UPS/XPS/KPFM, extraction method stated); illuminated *J*–*V* (spectrum, intensity, area); PL quenching or TA/TRPL lifetime change (excitation conditions); hysteresis/stability under cycling	104–112
Photodetection (high *R*, high D*)	Type-II + field-driven separation, or interlayer/photogating-assisted separation (often trap-kinetics limited)	SnSe_2_/MoS_2_ vertical heterostructure; MoS_2_/MoSe_2_ vertical device; GaN/MoS_2_ vdW junction; CVD MoS_2_/h-BN; MoS_2_/WS_2_ with hole transfer	*R* and D* (noise model/NEP and calculation stated); rise/decay time (fit protocol, instrument limit); dark *vs.* illuminated *I*–*V*; spectral response (wavelength range); test bias, optical power density, spot size/modulation frequency	8, 18, 30, 49 and 51–54
FET/contact-dominated MoS_2_ devices	Low Schottky barrier; depinned or 1D/edge-like contact; clean vdW interface (often FLP/*ρ*_c_ limited)	MoS_2_/rubrene vdW FET; photovoltaic FET based on MoS_2_/rubrene; super-aligned CNT contact to MoS_2_; Au/Pd–MoS_2_ contact with known SBH; CVD-grown MoS_2_/h-BN vertical stack used as contact platform	SBH or contact resistivity (TLM or temperature-dependent *I*–*V*, method stated); *µ*_FE_ (two-/four-probe, extraction stated); *I*_on_/*I*_off_ and gate-bias range; sweep rate and hysteresis (environment specified)	30, 81, 82, 85 and 86
HER/electrocatalytic MoS_2_ (interface activation)	Interface-induced Fermi-level/phase/strain modulation; well-coupled junction; ion-permeable surface (often *R*_ct_/stability limited)	MoO_2_/MoS_2_ vertical heterostructure (space-confined growth); GQD-assisted exfoliated 0D/2D MoS_2_ HER heterojunction	*η*_10_ at 10 mA cm^−2^ (electrolyte, pH, iR correction); Tafel slope; EIS *R*_ct_ (before/after cycling); durability protocol (cycle number/time) and post-test structure (optional)	113 and 114
Energy storage/hybrid capacitive devices	Ion-permeable, strain-accommodating interface; continuous conductive skeleton (often ion/electron transport limited)	3D MXene/MoS_2_ heterostructure with shielding MoS_2_ layer; TBA-intercalated 1T-MoS_2_ vertically coupled on delaminated MXene; MoS_2_/carbon composites	Rate/areal capacity (current density/scan window stated); retention after *N* cycles (protocol stated); EIS before/after cycling; electrode loading/thickness; post-mortem structure (SEM/XRD/Raman, optional)	48, 50, 62, 71 and 115
Photocatalysis/PEC	Favourable band bending or Type-II/direct Z-scheme separation at the MoS_2_ interface (often surface recombination limited)	Mesoporous black TiO_2_/MoS_2_/Cu_2_S tandem; BiVO_4_/MoS_2_; PCN-224@MoS_2_ direct Z-scheme; MoS_2_/Bi_2_O_3_; TiO_2_/MoS_2_	Photocurrent or product rate (electrolyte, pH, potential window stated); PL/EIS evidence (same test conditions); light source (spectrum, intensity, area) and stability duration; faradaic efficiency if applicable	32, 48, 71 and 116–119

The starting point of this route is the device-level figure of merit rather than a preselected material pair. In practice this may be the power conversion efficiency and *V*_oc_ for MoS_2_-based or 2D/3D photovoltaic junctions, the responsivity *R* and specific detectivity D* for van der Waals photodetectors,^8,18,103,120,121^ the overpotential at 10 mA cm^−2^ and the Tafel slope for MoS_2_/oxide or MoS_2_/sulfide HER electrocatalysts,^113,122–126^ or the ratio *I*_on_/*I*_off_ together with the contact resistance for MoS_2_ transistors and contact-engineered devices.^17,18,86,87,90^ Each of these metrics is limited by a dominant loss channel such as interfacial recombination, trap capture, insufficient carrier or ion transport, or current controlled by the contact, so the interface to be engineered has to address that specific loss.

Once the target metric has been identified, it should be reformulated as an interfacial mechanism that can be verified experimentally. Photoactive devices typically require a Type-II band alignment to spatially separate photo-generated carriers, together with a built-in electric field provided by a Schottky or asymmetric contact to drive the carriers across the junction and suppress non-radiative recombination.^52,68,69,76^ Electrochemical and energy-storage systems, by contrast, benefit from Fermi-level tuning, charge- or strain-induced redistribution, the creation of phase or Mott–Schottky boundaries, or ion-permeable junctions, because these features accelerate charge-transfer kinetics and keep active sites accessible.^62,115,123,124^ Devices whose performance is controlled by the contact require low-barrier, depinned interfaces, which has been shown for Au, Pd, Ni, Ag and 1D edge contacts to MoS_2_.^86–89^ At this stage the requirement should be stated in quantitative terms, for example as the conduction- and valence-band offsets Δ*E*_c,v_, the Schottky barrier height *Φ*_B_, the amount of PL quenching or the reduction of transient-absorption lifetime, the charge-transfer resistance *R*_ct_ from EIS, or the mobility and contact resistivity extracted using a standard protocol.^52,53,127^ Writing the requirement in this way makes reports on different MoS_2_ heterostructures directly comparable.

After the interfacial requirement has been fixed, suitable MoS_2_-based heterostructure pairings and device geometries can be selected in a targeted manner. Interfaces that must provide a combination of Type-II alignment and built-in field can be realised by vertical MoS_2_/TMD stacks such as MoS_2_/WSe_2_ or MoS_2_/WS_2_, or by MoS_2_/Si and MoS_2_/InP two-dimensional–three-dimensional junctions with asymmetric contacts.^8,18^ Interfaces that are required to offer Fermi-level modulation together with ion accessibility and strain accommodation can be implemented by 1T/2H-MoS_2_ phase junctions, by MoS_2_/CoS_2_ or MoS_2_/MoP heterostructures, or by vertically coupled MoS_2_/MoO_2_ grown in confined space for alkaline HER.^113,122,124–126^ Where the contact barrier is the dominant bottleneck, MoS_2_/h-BN/metal, MoS_2_/graphene/metal or super-aligned CNT–MoS_2_ contacts are appropriate choices.^85,87,114^ In all cases the geometry, whether vertical or lateral and whether planar or core–shell, should be determined at the same time as the material combination, because it controls how effectively the intended interfacial coupling acts over the real device area.^23,33–35^

In this way the interface is no longer chosen by trial and error, but is traced back to the device metric it is meant to improve and to the specific interfacial physics that can deliver it. The applications discussed in the next section can therefore be read as case studies of this route, with their reported measurements showing how completely the intended coupling was achieved.

## 4 Mapping interfacial coupling to device performance: photovoltaics and energy storage

This chapter discusses photovoltaic and energy-storage applications of MoS_2_-based heterostructures from a mechanism-to-metrics perspective. We emphasise how shared interfacial coupling modules translate into distinct device pathways and performance trade-offs under different application targets, linking key KPIs to the corresponding interface-engineering levers. To improve clarity beyond case-by-case reporting, each subsection ends with a comparative insight summarising quantitative trends and critical distinctions between heterostructure types.

### 4.1 Applications of MoS_2_-based heterostructures in photovoltaic devices

Following the mechanism-to-metrics perspective outlined at the beginning of this chapter, photovoltaic (PV) implementations of MoS_2_-based heterostructures are discussed by mapping target device metrics to interfacial coupling requirements and then to practical pairing/geometry and interface-engineering levers. The primary PV targets are PCE and its components (*V*_OC_, *J*_SC_ and FF), together with practically decisive metrics such as hysteresis, operational stability and bias-/area-normalised leakage. These targets generally trace back to three coupled interfacial requirements that recur across different stacks: an energetically favourable separation landscape (staggered offsets and/or depletion-assisted built-in fields), a kinetic advantage of separation/extraction over recombination, and contacts that do not override junction physics *via* large *R*_c_ or partial Fermi-level pinning (FLP). In this sense, similar nominal “Type-II” band diagrams can produce distinct PV outputs because the rate-limiting segment may localise at the junction (recombination/traps), along the transport pathway (blocking barriers) or at the contacts (contact-limited extraction), leading to different KPI trade-offs.

**Fig. 7:**
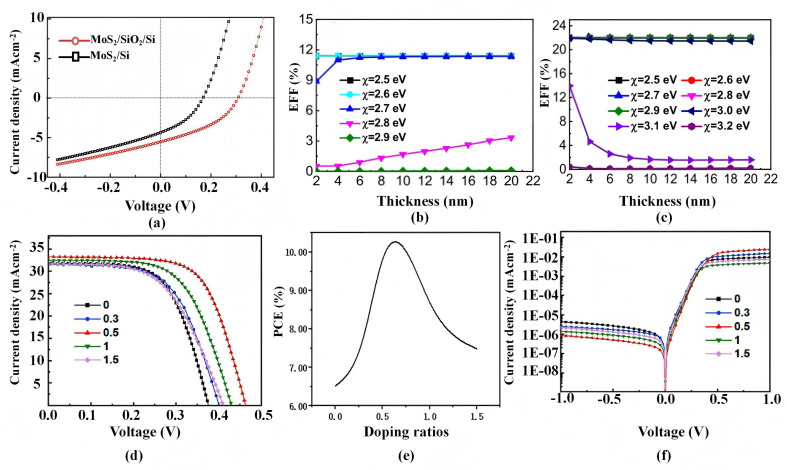
Quantitative examples showing how interface and contact engineering translate coupling requirements into photovoltaic KPIs in MoS_2_-based heterostructures. (a) Photovoltaic characteristics of devices with and without a SiO_2_ buffer layer under 15 mW cm^−2^ illumination; the buffered device shows a relative PCE improvement compared with the device without SiO_2_.^128^ Reproduced from ref. 128 with permission from the Royal Society of Chemistry. (b and c) Simulated PCE variation with MoO_*x*_ thickness and electron affinity for p-MoS_2_/p-MoO_*x*_/n-Si and p-MoO_*x*_/p-MoS_2_/n-Si solar cells, respectively, illustrating placement-dependent barrier shaping; positioning MoO_*x*_ between MoS_2_ and the metal electrode yields efficiencies above 20% in the reported simulation, whereas insertion at the MoS_2_/Si interface can block electron transport and reduce performance.^129^ Reproduced from ref. 129 with permission from IOP Publishing. All rights reserved. (d–f) MoS_2_ thin-film cells with different thiourea-doping ratios.^130^ Reproduced from ref. 130 with permission from Elsevier, copyright 2022. (d) *J*–*V* characteristics under illumination. (e) PCE as a function of thiourea-doping ratio, with an optimum near a ratio of 0.5. (f) Semi-logarithmic dark *J*–*V* curves showing leakage/rectification changes with doping ratio.

Historically, early PV demonstrations concentrated on MoS_2_/Si heterojunctions, where a depletion-assisted built-in field provides a direct driving force for photocarrier separation and enables preliminary photoelectric conversion.^131^ As the field matured, the focus shifted from merely forming a junction to controlling where the built-in field and barriers reside and suppressing recombination along the intended extraction pathway. One widely adopted approach is interfacial buffering/passivation, for example inserting an ultrathin SiO_2_ layer to reduce interfacial trap-assisted loss. The resulting pathway improvement is reflected in the illuminated *J*–*V* characteristics and the PCE increase reported for buffered MoS_2_/Si devices (Fig. 7(a)).^128^ Importantly, such buffering is beneficial only when it primarily suppresses recombination/leakage while preserving efficient carrier transmission (*e.g.*, *via* tunnelling-permissive thickness), thereby improving *V*_OC_ and FF without sacrificing *J*_SC_.

Once junction recombination is mitigated, many MoS_2_/Si PV stacks become increasingly sensitive to contact-related bottlenecks, making selective-contact barrier shaping a decisive lever for FF and practical PCE. This is clearly illustrated by MoO_*x*_ interlayers, where the placement of the modifier determines whether it strengthens the desired pathway or creates a new transport barrier. Simulations indicate that inserting MoO_*x*_ between MoS_2_ and the metal electrode can raise the predicted efficiency to above 20% by improving selectivity and reducing contact-induced losses (Fig. 7(b) and (c)).^129^ In contrast, placing MoO_*x*_ at the MoS_2_/Si junction suppresses electron transport and drastically reduces performance.^129^ This placement dependence directly supports the chapter-level mapping logic: the same interfacial “mechanism” (barrier modification) can either enhance or degrade PV KPIs depending on whether it suppresses the dominant bottleneck segment without introducing blocking barriers along the extraction route.

In parallel, chemical/defect-state modulation offers another route to shift the rate-limiting physics by simultaneously tuning trap densities, transport barriers and film stability. For example, solution-processed thiourea doping has been reported to improve crystallinity and interfacial stability of MoS_2_ thin films.^130^ In n-MoS_2_/p-Si heterojunctions, an optimal thiourea-doping ratio yields a peak efficiency of 9.81%, accompanied by strengthened rectifying behaviour and reduced dark leakage (Fig. 7(d)–(f)).^130^ From the mechanism-to-metrics viewpoint, the optimum reflects a net gain where trap-assisted recombination and parasitic leakage are suppressed more strongly than any mobility loss or barrier increase introduced by dopant-related chemistry, thereby improving the balance between separation/extraction and recombination.

As the same interfacial modules are translated to other material pairings, MoS_2_ integration has expanded beyond silicon to III–V semiconductors, II–VI compounds and layered partners, with performance again determined by whether field localisation, recombination suppression and contact management are realised in the device pathway. Although n-MoS_2_/p-InP devices initially show modest efficiencies, they validate compatibility with high-mobility platforms and motivate further interface/contact optimisation.^132^ MoS_2_/CdS heterostructures fabricated by chemical-bath deposition form stable thin-film junctions with favourable *I*–*V* characteristics and photovoltaic response, illustrating that scalable processing can yield viable PV pathways provided that interfacial recombination is controlled.^105^ In van der Waals stacks, vertically assembling MoS_2_ with other layered semiconductors and graded-alloy partners can broaden spectral utilisation and enhance separation selectivity; however, whether these gains translate into higher PCE still hinges on the same pathway constraints (low-loss extraction and suppressed parasitic channels) rather than on nominal offsets alone.

Moreover, MoS_2_ is widely incorporated as a functional interfacial component within composite PV architectures, where its most consistent contribution is to tune recombination and extraction selectivity. In graphene-based, perovskite and transparent solar cells, MoS_2_ has been used as an electron-transport layer (ETL), hole-transport layer (HTL) or passivation/interfacial modifier, often improving efficiency and stability by suppressing trap-mediated loss and hysteresis.^108–111,133^ Representative demonstrations include MoS_2_/graphene/n-Si structures reaching 11.1% conversion efficiency and MoS_2_/h-BN/GaAs heterojunctions achieving 9.03% under interfacial modulation,^10,108,134^ consistent with the roles of contact selectivity and vdW decoupling in reducing recombination and contact-induced limitations. Incorporating MoS_2_ into perovskite solar cells has further raised efficiencies to above 13%, where MoS_2_ typically functions as a coupling modifier that passivates interfacial defects and stabilises interfacial energetics rather than serving as the primary absorber. Simulation studies also suggest that MoS_2_/c-Si heterojunctions can exhibit higher quantum efficiency in the short-wavelength region than conventional a-Si/c-Si architectures, highlighting their potential in tandem and multijunction designs where spectral utilisation and interface loss control are both critical.^135^

Overall, PV research on MoS_2_-based heterostructures has evolved from establishing simple MoS_2_/Si junctions to pathway-oriented designs that emphasise field localisation, recombination suppression and contact/barrier management. Fig. 7 anchors this mapping with quantitative examples, showing how buffering/passivation, selective-contact interlayers and dopant-driven defect/transport modulation translate interfacial coupling requirements into measurable PV KPIs. Viewed comparatively, unbuffered Schottky-like junctions are commonly limited by interfacial recombination and leakage (constraining *V*_OC_ and FF), whereas ultrathin buffers or vdW spacers improve photovoltage and leakage by suppressing trap-assisted loss; once a usable junction is formed, further gains increasingly depend on preventing contact-induced barriers and parasitic bypass paths from dominating the measured *J*–*V* response (thereby limiting FF). This diagnosis-first logic (identify the dominant bottleneck → select pairing/geometry and interlayer placement accordingly) operationalises the chapter theme of mapping interfacial coupling to device performance.

### 4.2 Applications of MoS_2_-based heterostructures in energy storage

In energy-storage devices, MoS_2_ is attractive because its layered framework offers ion-accessible pathways and abundant electrochemically active sites. In practice, however, pristine MoS_2_ electrodes are frequently limited by two coupled bottlenecks: insufficient electronic conductivity and structural/chemical instability under repeated cycling (restacking, volume change, and interfacial degradation). Consistent with the mechanism-to-metrics logic adopted in this chapter, the key energy-storage targets—high specific/areal capacity (or capacitance), high-rate capability, long-cycle retention, and suppressed impedance growth (*e.g.*, reduced *R*_ct_ and stable EIS signatures)—can be traced back to three recurring interfacial requirements: maintaining ion-accessible transport channels, establishing continuous electron percolation networks, and stabilising the interface against mechanical and chemical evolution. Recent MoS_2_-based heterostructures address these requirements through conductive coupling, strain-accommodating architectures, and multifunctional interfacial composites, thereby translating interfacial engineering into measurable electrochemical KPIs.

A direct route to improving rate performance and cycling stability is to couple MoS_2_ with redox-active or pseudocapacitive partners that enhance charge-storage kinetics while preserving an ion-accessible interface. For instance, a MoS_2_/MnO_2_ nanosheet heterostructure prepared *via* an electrochemical *in situ* route delivered a specific capacitance of 275 Fg^−1^, exceeding that of pure MoS_2_, and maintained excellent cycling stability in neutral electrolytes over 10 000 cycles at high current density.^136,137^ From a coupling perspective, the performance gain is consistent with a heterointerface that provides additional faradaic contribution and improved interfacial kinetics, while sustaining ion accessibility and mitigating degradation during long-term cycling. Related architecture-level optimisation can further shift the rate-limiting segment from bulk transport to interfacial kinetics, as illustrated by hydrothermally synthesised MoS_2_ nanoworm aggregates used in an asymmetric supercapacitor, which achieved an energy density of 103.51 Wh kg^−1^ and a power density of 3807.59 W kg^−1^, retaining >93% capacity over long cycling.^138^ Here, the key mapping is that hierarchical morphology preserves electrolyte access and short diffusion pathways, enabling simultaneously improved energy/power output and retention.

When the dominant bottleneck is electronic transport and electrode integrity at high current density, introducing conductive scaffolds is typically the most effective lever. MoS_2_–carbon composite heterostructures exemplify this mechanism-to-metrics translation: by providing continuous electron percolation while suppressing MoS_2_ restacking and preserving active surface area, these hybrids can deliver high capacity with improved durability. For example, MoS_2_/carbon hybrid electrodes reported an initial capacity >920 mAh g^−1^ in Li-ion batteries and retained ∼88% capacity after 500 cycles.^139,140^ Interpreted through pathway control, the improved retention suggests that the heterointerface maintains electrical connectivity and mitigates pulverisation/SEI-driven impedance growth, which would otherwise rapidly erode the effective utilisation of MoS_2_ at prolonged cycling.

Beyond conventional Li-ion batteries and supercapacitors, MoS_2_-based heterostructures have been extended to emerging energy-storage chemistries where interfacial coupling is explicitly used to tune ion transport and reaction kinetics. A mixed-phase 1T/2H MoS_2_/g-C_3_N_4_ heterostructure enabled efficient aqueous NH_4_^+^ storage with a specific capacitance of 426 Fg^−1^ and 93.9% capacity retention after 45 000 cycles, combining ultra-long lifetime with environmental friendliness.^141^ In mapping terms, phase-enabled conductivity (1T contribution) and a stable, ion-accessible heterointerface are jointly required to sustain fast kinetics over extremely long cycling. In a related direction, a MoO_3_–MoS_2_ composite heterostructure was developed as a biodegradable energy-storage unit that integrates supercapacitor and disposable-battery functions; its Zn-ion hybrid supercapacitor achieved an energy density of 30.56 µWh cm^−2^ and operated stably in both air and liquid.^142^ This example highlights that for emerging device scenarios (*e.g.*, bio-integrated electronics), interface stability and compatibility with operating environments can become a primary KPI alongside energy density. Moreover, under practical high-loading conditions where transport limitations become severe, multiphase-interface control can be used to lower diffusion barriers and preserve effective areal performance. A Mo_2_C–MoN@MoS_2_ multi-heterostructure reduced the Li^+^ diffusion energy barrier and increased areal capacity to 9.6 mAh cm^−2^ under high loading,^143^ consistent with a pathway optimisation where rapid ion transport and robust electronic coupling are engineered at multiphase interfaces to avoid “dead-zone” formation in thick electrodes.

In addition to energy storage alone, MoS_2_ heterostructures are increasingly explored in multifunctional energy-related systems, where the mapping logic remains useful for separating the roles of storage, stability and auxiliary functions. A MoS_2_–a-SiC heterostructure delivered a 1.5× increase in specific capacitance and improved stability over >4000 cycles as a supercapacitor electrode, while also enabling stable detection of high-concentration NO_2_ at elevated temperatures.^144^ Although the sensing function is distinct, the storage improvement is still consistent with enhanced interface robustness and maintained transport pathways under harsh conditions. Similarly, a Ge-decorated h-BN/MoS_2_ heterostructure achieved a high capacitance of 558.53 Fg^−1^ and also showed low overpotentials with good stability for the hydrogen-evolution reaction (HER),^145^ illustrating how heterointerface design can couple ion/electron transfer benefits across storage and catalysis. Finally, for Li–S batteries where parasitic shuttle and interfacial side reactions dominate degradation, heterostructure interfaces can be designed as “chemical traps + catalytic conversion” pathways. A MoS_2_/Co_4_S_3_/C hollow heterostructure suppressed the polysulfide shuttle *via* synergistic adsorption and catalysis, achieving a capacity-decay rate of only 0.04% after 1 000 cycles,^146^ emphasising that in such systems the decisive KPI is long-cycle stability enabled by controlling interfacial parasitic pathways rather than by maximising initial capacity alone.

Overall, recent progress shows that MoS_2_-based heterostructures improve energy-storage performance by converting intrinsic active sites into stable, ion-accessible and electronically percolated pathways. Conductive coupling most directly enhances rate capability and mitigates cycling fade by maintaining electron transport continuity and suppressing restacking, whereas morphology/architecture engineering primarily preserves ion accessibility and reduces diffusion length, enabling higher power output without sacrificing retention. Multiphase or multifunctional hybrids can further raise capacity or enable specialised operating scenarios, but they are also more sensitive to interfacial side reactions and structural evolution, making retention, impedance growth and coulombic efficiency critical discriminators beyond peak values. These comparative trends reinforce the chapter theme: the rational selection of pairing/geometry should follow from diagnosing which segment of the ion-electron pathway is rate-limiting (ion accessibility, electron percolation, or interface stability) and then applying the corresponding interface-engineering lever to improve the targeted energy-storage KPIs.

## 5 Conclusions and outlook

Interface engineering in MoS_2_-based heterostructures has become a central route to push two-dimensional devices beyond the intrinsic limits of a single material. By consolidating construction strategies, material-pairing spaces and the recurring interfacial physics that governs real devices, this review highlights that performance is rarely determined by nominal band alignment alone, but by the effective interfacial energy landscape, kinetic competition among charge transfer, recombination and trapping, and the constraints imposed by contacts and parasitic pathways.

A key message of this work is a target-metrics-driven design logic that links device figures of merit to measurable interfacial descriptors and then to actionable engineering levers. Instead of starting from a preferred material pair, the recommended route is to back-trace from the target metric to a set of interfacial requirements—band offsets and built-in fields, dipole steps, the rate hierarchy among *k*_CT_, *k*_rec_ and *k*_trap_, acceptable trap densities, and an upper bound on contact resistance *R*_c_—and then select pairing and configuration to realize these constraints under realistic processing conditions. Within this view, vertical, lateral and mixed-dimensional configurations are best treated as choices of pathway topology and non-ideality localization, while pairing, geometry, passivation/encapsulation and contact engineering provide the practical knobs to enforce the desired pathway and suppress bypass channels. Importantly, the same framework also implies a minimum reporting checklist for cross-study comparability: interfacial band/built-in-field descriptors, kinetic evidence for the intended rate hierarchy, trap-related metrics linked to hysteresis or slow transients, and quantitative *R*_c_ together with leakage/shunt diagnostics.

For photovoltaics and photodetection, achieving efficient exciton dissociation and directional carrier extraction requires that the effective junction field and transfer pathways allow *k*_CT_ to outcompete non-radiative loss channels, while contact and edge leakage must be controlled to prevent pathway dilution. For energy-related electrochemical systems, the decisive interface attributes shift to ion-accessible and strain-accommodating junctions with stable charge-transfer pathways, where long-term interfacial chemistry and structural evolution often dominate the practical lifetime metrics.

Looking forward, several bottlenecks must be resolved for manufacturable technologies. Wafer-scale synthesis with controlled defect statistics and clean interfaces remains essential, motivating transfer-free growth and stacking routes that preserve interfacial integrity. Quantitative interface metrology under operating conditions is needed to directly determine band offsets, dipole fields, charge-transfer dynamics and local strain, enabling mechanistic validation rather than qualitative assignment. Interface stability must be engineered alongside electronic functionality through encapsulation and chemical control that maintain the intended coupling while suppressing environmental sensitivity. Finally, tighter theory–data synergy is required: predictive screening should be paired with interoperable experimental datasets and standardized reporting so that models can be trained, falsified and refined on consistent descriptors. With these advances, MoS_2_-based heterostructures are well positioned to evolve from proof-of-concept demonstrations toward reproducible, scalable and application-ready energy technologies.

## Author contributions

Linhou Cong and Weisheng Yang contributed equally to this work. Linhou Cong: conceptualization; methodology; investigation; data curation; software; visualization; writing – original draft; writing – review & editing. Weisheng Yang: conceptualization; supervision; project administration; funding acquisition; validation; writing – review & editing. Zixuan Yan: investigation; data curation; writing – review & editing. Siyu Chen: resources; writing – review & editing. Peijin Yang: validation; writing – review & editing. All authors reviewed and approved the final manuscript.

## Conflicts of interest

There are no conflicts to declare.

## Data Availability

No primary research results, software or code have been included and no new data were generated or analysed as part of this review.
